# Editorial: The stem cell niche in brain aging and neurodegenerative disorders

**DOI:** 10.3389/fnagi.2026.1969100

**Published:** 2026-09-08

**Authors:** Shouneng Peng, Vanessa Castelli, Gangcai Xie, Lilach Soreq

**Affiliations:** 1 Jinggangshan University, Jiangxi, China; 2 University of L'Aquila, L'Aquila, Italy; 3 Nantong University, Nantong, China; 4 University College London, London, United Kingdom

**Keywords:** adult neurogenesis and cell biology, brain aging, neural stem cell, microglia, neurodegenerative diseases (ND), stem cell niche

As the global population ages, late-life neurodegenerative diseases, such as Alzheimer's (AD) (Peng et al., 2021) and Parkinson's disease (PD), pose a mounting challenge to healthcare and society. A critical, yet historically underappreciated, element in understanding brain aging and the progression of these diseases is the microenvironment surrounding neural stem cells (NSCs)—the stem cell niche. Throughout adulthood, specific niches within the brain, such as the subventricular zone (SVZ) and the subgranular zone (SGZ) of the hippocampus, sustain neurogenesis and gliogenesis (Elmore et al., 2018). These niches are not passive scaffolds; rather, vasculature, the extracellular matrix, resident immune cells, and trophic signaling together determine whether NSCs remain quiescent, proliferate, or differentiate. However, aging profoundly diminishes the regenerative potential of these environments. To illuminate this intricate landscape, the Research Topic brings together innovative research that explores the cellular, molecular, and microenvironmental shifts that dictate brain aging and neurodegeneration, along with advanced modeling strategies using *in vitro* and *in vivo* systems. This Research Topic features four compelling articles that collectively advance our understanding of how stem cells, glial cells, and their modulators respond to and drive the aging process.

## Niche identity and vulnerability

A fundamental question is how faithfully NSCs retain niche-specific identity once isolated, and how that identity responds to disease-relevant insults.

In one of the featured studies, Amartumur et al. addressed the challenge of studying the niche-associated cellular behaviors of adult NSCs by employing an engineered, microfluidics-based culture model. The authors directly compared the morphological and migratory characteristics of NSCs derived from the two primary adult neurogenic niches: the hippocampal SGZ and the ventricular SVZ. Their findings demonstrated that SVZ-derived NSCs exhibit longer neurites, increased motility, and dispersed migration patterns. Conversely, SGZ-derived NSCs displayed shorter neurites and a collective, radial migration pattern. By mapping these intrinsic morphological and migratory differences, this study not only highlights how distinct local microenvironments dictate NSC behavior but also establishes a valuable *in vitro* platform for future mechanistic disease modeling, such as that relevant to Alzheimer's pathology.

While stem cells act as the seed of regeneration, microglia—the brain's resident immune cells—act as the ever-vigilant gardeners of the niche. However, aging profoundly alters microglial function, shifting these cells away from homeostatic neuronal surveillance. Subhramanian et al. utilized *ex vivo* time-lapse imaging of acute brain slices to investigate microglial surveillance in the context of normal aging. Unexpectedly, they discovered that a subset of aged microglia loses its territorial confinement, adopting increased somatic mobility that closely resembles the migratory behaviors observed in reactive microglia in severe neurodegenerative conditions. Accompanied by prolonged and sustained calcium signaling, this emergence of mobile, partially activated microglial states highlights a profound adaptation to the aging milieu, suggesting that microglia shift their strategy to compensate for reduced process-mediated monitoring. These findings suggest that aging induces a “primed” phenotype in microglia, shifting their surveillance from process-directed monitoring to soma-mediated migration—a transition that likely alters the neurogenic niche's inflammatory tone and influences surrounding stem cell viability. This intermediate, primed glial state is directly relevant to the neuroinflammatory shift modeled by Amartumur et al., suggesting that glial priming may precede, and could lower the threshold for, more overt niche pathology.

## Harnessing the niche for functional restoration

Beyond local cellular interactions, the stem cell niche is heavily influenced by systemic and neuroendocrine factors, which also decline with age. Luo et al. explored this systemic axis by investigating the effects of administering Nerve Growth Factor (NGF) to an aging mouse model. They demonstrated that a single central injection of NGF effectively elevated key sex hormones for up to seven days. Notably, this systemic endocrine effect was linked to a localized increase in Gonadotropin-Releasing Hormone (GnRH)-immunoreactive cells and enhanced Sox2/GnRH co-expression in hypothalamic NSCs. This study provides compelling preclinical evidence that modulating specific growth factors within the niche could revitalize neuroendocrine function and mitigate age-related physiological declines.

These findings converge on a practical question: can niche biology be therapeutically engaged, and by what route? A systematic review by Habiba et al. addressed the translational potential of stem cell therapies, specifically evaluating the intranasal administration of stem cells and their derivatives for neurological and respiratory disorders. By synthesizing data from human clinical trials, the authors emphasized the viability of the intranasal route as a non-invasive, direct-to-brain delivery mechanism that effectively bypasses the blood-brain barrier. This comprehensive review outlines a promising avenue for translating basic stem cell research into accessible clinical interventions for complex neurodegenerative and systemic diseases.

## Broader context and future developments

Together, these contributions depict a stem cell niche that is at once a site of stable, origin-specific cellular identity; a target vulnerable to amyloid and inflammatory insults; a lever for restoring neurogenic and endocrine output via trophic signaling; and a candidate destination for cell-based therapeutics still awaiting rigorous clinical validation. The findings illustrate that the aging brain is not merely characterized by a linear loss of neurons, but by a dynamic and systemic failure of the microenvironments designed to protect and replenish them. Whether through the altered migration of adult NSCs, erratic mobility of aged microglia, or breakdown of neuroendocrine signaling, the stem cell niche proves to be a critical nexus of brain aging.

Looking ahead, the research presented here lays the groundwork for several exciting developments. The successful implementation of microfluidic chip devices and high-resolution spatial transcriptomics will soon allow researchers to map cell fate determinations within the niche with unprecedented resolution. As computational biology and artificial intelligence become further integrated into these workflows, we can anticipate more precise models (such as advanced brain-on-a-chip organoids) that faithfully mimic immune-driven brain aging. Furthermore, the optimization of non-invasive delivery methods, such as intranasal administration, will accelerate the translation of niche-targeted therapies from the bench to the bedside. Ultimately, targeting the stem cell niche, whether by rescuing resident microglial surveillance, rejuvenating the niche with exogenous growth factors, or delivering stem cell therapeutics non-invasively, offers a powerful, multifaceted strategy to delay cognitive decline and treat the root causes of neurodegenerative disorders.
